# Method of choosing soils as CEB or pavement layers based on geological and environmental information and mode of treatment^✰^

**DOI:** 10.1016/j.mex.2023.102407

**Published:** 2023-10-02

**Authors:** Manefouet Kentsa Bertille Ilalie, Kamgang Kabeyene Beyala Véronique, Katte Yato Valentine, Wouatong Armand, Nzenti Jean Paul, Ndjigui Paul Désiré, Elimbi Antoine

**Affiliations:** a Department of Earth Sciences, Faculty of Science, University of Dschang, West-Cameroon, Cameroon; b Department of Geology, Faculty of Science, University of Bukavu, Sud-Kivu, Democratic Republic of Congo; c Department of Civil and Environmental Engineering, University of Namibia, P.O. Box 3624 Ongwediva, Namibia; d Department of Earth Sciences, Faculty of Sciences, University of Yaoundé 1, Cameroon; e Department of Inorganic Chemistry, Faculty of Sciences, University of Yaoundé 1, Cameroon

**Keywords:** Specifications, Correlation-patterns, Compressed earth bricks, Pavement layers, Resistance, Durability, *Method of Choosing Soils, for treated/untreated CEBs/Pavement layers (CSCP method)*

## Abstract

During research on the geological, mineralogical, mechanical and geotechnical nature of thick layers of soils developed on the rocks of the lower zone of the southern slopes of Mount Bambouto (West Cameroon), a couple of observations were made. The mechanical behavior of soils as construction materials is linked to the nature of the parent rock and its environment of formation. Four different types of rock have been noted: trachybasalt, orthogneiss, anatexite, and biotite-hornblende granitoid. These rocks in a hot and humid mountain climate, exposed to the monsoon wind, have weathered to give soils with exploitable characteristics in compressed earth bricks (CEB) and pavement layers. This article proposes a method of choosing a soil for pavement layers and for the production of fired earth bricks / earth bricks treated or not with hydraulic binders, based on the nature of the bedrock and the climatic environment. Several formulation models have been defined. The performance, fairness, accuracy and adequacy of these models with real values were made by metric evaluation. So, it is:

• Preliminary method for quickly and easily formulating masonry units in clay soil.
• Preliminary method for quickly and easily formulating pavement layers in clay soil.
• Simple and reliable method for choosing clay soils as construction materials from elementary geological information.

## Graphical abstract

**Figure:**
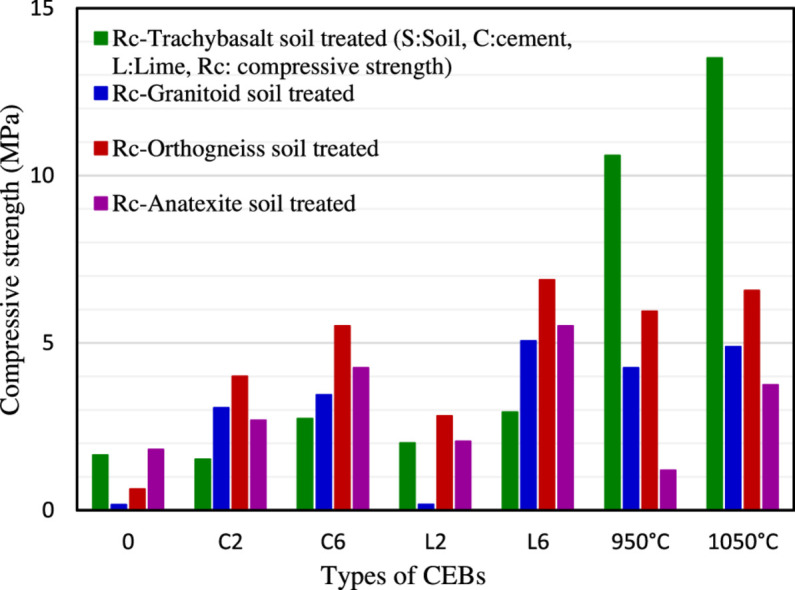

Some values of the compressive strength of CEBs depending on the nature of the parent rock, the binder dosage and the firing temperature.

Specifications Table

**Table:** 

Subject area:	Engineering
More specific subject area:	*Civil engineering, Geotechnics, Geology.*
Name of your method:	*Method of Choosing Soils, for treated/untreated CEBs/Pavement layers (CSCP method)*
Name and reference of original method:	*Not applicable*
Resource availability:	*Not applicable*

**Method details**

This paper proposes four charts (Fig. 1, Fig. 2, Fig. 3, Fig. 4) based on 4 patterns (Table 1, Table 2, Table 3, Table 4, Table 5, Table 6, Table 7, Table 8, Table 9) that allow a quick reading of the formulation of construction materials (treated or untreated earth bricks, pavement layers) according to the intended use of the structure and the environment of the structure. It also provides 36 correlations for determining the physical and mechanical characteristics (compressive strength, bending strength, water absorption rate) depending on the treatment adopted for the identified earth bricks. In addition, twelve correlations are presented to determine the CBR bearing index according to the dosage of binder or not, the number of days of immersion in water, or of air drying of the clay soil. All these correlations have a coefficient of determination between 0.91 and 1, except that (correlations 24 and 28) of the water absorption of CEB from lateritic clay soils developed on anatexite, depending on the lime dosage (R² = 0.6968) and that of the compressive strength of CEBs from lateritic clay soils developed on granitoid, depending on the cement dosage (R² = 0.8883) which are less than 1.

**Table 1:** Correlation context.

Factors	Pattern I	Pattern II	Pattern III	Pattern IV
Rocks	Trachybasalt	Granitoid	Ortogneiss	Anatexite
Duration (Myr)	]52,38[		]660,580[	
Orography	Semi-orange hills with steep slope and U-shaped talweg	Sharp summit, narrow thalweg and rocky escarpment	pointed summit and narrow thalweg	Steep, convex and concave slope
Weather	Average precipitation/year: 1830 mm; Temperature : from 14.8 °C to 25 °C			
Mineral and geochemical Combination	Min - Ox - I	Min – Ox - II	Min – Ox - III	Min – Ox - IV

*Note:* Min. = minerals; Ox = oxides.

**Table 2:** Pattern I: Statistics of soil oxides and minerals (in weight-%) developed on trachybasalt.

%	Max.	Min.	Average	S.D.	Minerals	Max.	Min.	Average	S.D.
SiO_2_	32.56	0.43	12.64	14.22	Gibbsite	60.89	10.22	22.30	27.27
TiO_2_	8.38	2.62	5.99	2.57	Kaolinite	86.92	5.63	26.75	44.61
Al_2_O_3_	43.71	12.53	27.64	12.99	Quartz	0.7	0.18	0.22	0.37
Fe_2_O_3_	67.26	18.25	39.36	21.42	Anatase	9.1	3.1	4.75	3.03
MnO	0.11	0.05	0.07	0.02	Goethite	84.15	18.09	40.56	33.43
MgO	0.53	0.00	0.23	0.22	Hematite	12.93	12.93	3.23	
CaO	0.00	0.00	0.00	0.00	Magnetite	4.81	3.98	2.20	0.59
Na_2_O	0.01	0.00	0.01	0.00					
K_2_O	0.02	0.00	0.01	0.01					
P_2_O_5_	0.66	0.26	0.46	0.23					
L.I.	20.35	12.15	15.33	3.51					

*Note:* Max: Maximum Min: Minimim; SD: Standard Deviation; L.I.: Lost of Ignition.

**Table 3:** Pattern II: Statistics of soil oxides and minerals (in weight-%) developed on biotite and hornblend granitoid.

%	Max.	Min.	Average	S.D.	Minerals	Max.	Min.	Average	S.D.
SiO_2_	64.51	55.51	59.70	4.082	Kaolinite	31.83	8.6	15.63	11.67
TiO_2_	2.09	0.72	1.16	0.629	Quartz	40.4	21.38	30.33	8.03
Al_2_O_3_	21.59	14.83	18.25	2.993	Magnetite	1.93	1.93	0.48	–
Fe_2_O_3_	12.05	5.30	7.65	3.133	Muscovite	15.25	7.5	10.56	3.64
MnO	0.14	0.03	0.08	0.044	Biotite	34.8	10.87	11.42	16.92
MgO	5.10	0.53	2.21	1.991	Plagioclase	11.51	11.51	2.88	–
CaO	1.93	0.00	0.48	0.963	Microcline	36.96	9.9	27.87	12.75
Na_2_O	1.80	0.01	0.46	0.896	Actinolite	3.29	3.29	0.82	
K_2_O	6.12	2.35	4.19	1.565			–		
P_2_O_5_	0.18	0.03	0.08	0.066					
L.I.	8.37	3.65	5.83	2.134

**Table 4:** Pattern III: Statistics of soil oxides and minerals (in weight-%) developed on orthogneiss.

%	Max.	MinN	Average	S.D.	Minerals	Max.	Min.	Average	S.D.
SiO_2_	64.74	60.21	64.74	17.95	Quartz	47.56	47.56	47.56	–
TiO_2_	0.42	0.01	0.42	0.28	kaolinite	43.07	43.07	43.07	–
Al_2_O_3_	21.00	0.01	21.00	11.29	Gibbsite	9.37	9.37	9.37	–
Fe_2_O_3_	3.44	0.02	3.44	1.97			–		
MnO	0.01	0.00	0.01	0.02					
MgO	0.08	0.00	0.08	0.16					
CaO	0.00	0.00	0.00	0.55					
Na_2_O	0.01	0.01	0.01	1.52					
K_2_O	0.12	0.00	0.12	2.21					
P_2_O_5_	0.03	0.00	0.03	0.03					
H_2_O	9.59	0.18	9.59	4.99

**Table 5:** Pattern IV: Statistics of soil oxides and minerals (in weight-%) developed on anatexite.

%	Max.	Min.	Average	S.D.	Minerals	Max.	Min.	Average	S.D.
SiO_2_	68.25	59.58	63.91	6.13	Gibbsite	23.47	21.25	22.36	1.57
TiO_2_	1.45	0.80	1.13	0.46	Kaolinite	26.3	18.56	22.43	5.47
Al_2_O_3_	19.02	15.50	17.26	2.49	Quartz	54.54	52.47	53.51	1.46
Fe_2_O_3_	10.56	6.31	8.43	3.01	Hematite	3.43	3.43	1.72	–
MnO	0.04	0.02	0.03	0.01			–		
MgO	0.05	0.03	0.04	0.01					
CaO	0.00	0.00	0.00	0.00					
Na_2_O	0.01	0.01	0.01	0.00					
K_2_O	0.14	0.03	0.08	0.08					
P_2_O_5_	0.12	0.04	0.08	0.06					
L.I.	9.35	7.98	8.67	0.97

**Table 6:** Average CBR (%) values of soils treated and untreated lateritic soils.

Soil treated with cement (x for correlation)	Cement-Tb	Cement-An	Soil treated with lime (x for correlation)	Lime-Tb	Lime-An
	Pattern I	Pattern IV		Pattern lI	Pattern IV
	CBR (%)			CBR (%)	
S - 0	43	18	S - 0	43	18
S – C – 2 - 4	43	30	S – L – 2 - 4	60	53
S – C – 2 - 7	74	55	S – L – 2 - 7	82	72
S – C – 2 - 14	94	80	S – L – 2 - 14	94	92
S – C – 4 - 4	90	42	S – L – 4 - 4	90	92
S – C – 4 - 7	126	60	S – L – 4 - 7	126	113
S – C – 4 - 14	153	80	S – L – 4 - 14	153	133
S – C – 6 - 4	108	35	S – L – 6 - 4	82	70
S – C – 6 - 7	133	55	S – L – 6 - 7	140	100
S – C – 6 - 14	163	72	S – L – 6 - 14	180	135

*Note:* C = cement, *L* = lime, *S* = Soil, Tb : trachybasalt, An : anatexite, S-0 = natural clay soil.

S-L-i-j or S-C-i-j: "Clay soil (S) treated with (i)% of lime (L) or cement (C) after (j-4) days air curing, and 4 immersion days. e.g.: S-C-6–14: Clay soil treated with 6 % of cement after 10 days air curing, and 4 immersion days.

**Table 7:** Characteristics of soil for pavement layers.

Physical parameters	Pattern I		Pattern IV	
	Reddish lateritic soil on trachybasalt	Nature of soil	Dark red clay soil on anatexite	Nature of soil
d/D	0/25	Texture gravelo-argileuse, sol majoritairement grenu (74%)	0/4	Balanced sandy-clayey texture, grainy and finely balanced soil
$D_{10}$ (mm)	0		0	
$D_{30}$ (mm)	0.08		0	
$D_{60}$ (mm)	9.50		0.17	
$C_{c}$	→∞	Poorly graded soil	→1	Poorly graded soil
$C_{u}$	→∞	Spread-grained soil (varied)	→∞	Spread-grained soil (varied)
FM	3.50	Ground with fine character	1.14	Soil with less fine character
$\gamma_{s}$ (kN/m^3^)	27.80	Soil rich in secondary minerals (kaolinite)	26.50	Soil rich in primary minerals (quartz)
LL (%)	55.20	–	74.45	$\omega_{l}>70\Rightarrow$ soil at risk
PL (%)	37.30	–	38.70	–
PI (%)	18	More or less plastic soil	35.75	Plastic soil
CI	2.25	Hard soil	1.43	Hard soil
$\omega_{nat}\left(\%\right)$	14.70	–	23.40	
$\gamma_{dMPO}$(kN/m^3^)	20.00	–	17.64	–
$\omega_{OPM}\left(\%\right)$	13.30	–	18.20	–
$CBR_{95{\%}_{MPO}}$	43.00	Bearing class soil $S_{5}$	18	Bearing class soil$\;S_{4}$
H.R.B. Class	A-2–7 (2)	Acceptable as road basement	A-7–5 (12)	Bad as road basement
	Use as pavement layers

**Table 8:** Physical and mechanical properties of CEB of various patterns (I, II, III and IV).

Types of CEB	Volumic mass (**g/cm^3^**)				Absorption (Abs) of water (g/cm^2^.min^1/2^) of CEB			
	Pattern I	Pattern II	Pattern III	Pattern IV	Pattern I	Pattern II	Pattern III	Pattern IV
0	1.74	1.90	2.00	2.02	–	–	–	–
C2	1.61	1.89	1.99	1.95		1.26	1.43	1.20
C4	1.66	1.92	1.97	1.98	2.59	1.17	1.29	1.16
C6	1.69	1.90	1.97	1.96	2.60	1.12	1.27	1.16
C8	1.69	1.88	1.99	1.96	2.44	1.08	1.20	1.20
L2	1.67	1.86	1.96	1.99	–	–	–	1.14
L4	1.67	1.89	1.99	2.01		1.26	1.20	1.30
L6	1.62	1.88	2.00	1.99	2.67	1.25	1.26	1.21
L8	1.69	1.89	1.99	1.99	2.39	1.20	1.10	1.16
950 °C	1.50	1.79	1.73	1.67	2.01	1.35	1.34	0.94
1050 °C	1.46	1.77	1.73	1.71	2.14	1.30	1.35	0.94
1100 °C	1.56	1.79	1.73	1.72	1.93	1.29	1.30	0.90

Types of CEB	Compressive strength (MPa) of CEB				Bending strength (MPa) of CEB			
	Pattern I	Pattern II	Pattern III	Pattern IV	Pattern I	Pattern II	Pattern III	Pattern IV

0	1.65	0.16	0.63	1.81	0.31	0.33	0.33	0.3
C2	1.52	3.06	4	2.69	0.52	0.6	0.6	0.66
C4	2.32	3.31	4.88	3.06	0.8	1.27	1.27	1.15
C6	2.74	3.44	5.5	4.25	0.91	1.44	1.44	1.41
C8	3.05	4.06	5.94	5.13	1.52	1.99	1.99	1.76
L2	2.01	0.17	2.81	2.06	0.42	0.66	0.66	0.58
L4	2.32	3.19	2.94	3.31	0.68	1.23	1.23	1
L6	2.93	5.06	6.88	5.5	0.92	2.13	2.13	1.56
L8	3.54	6.31	8.44	5.88	1.26	2.1	2.1	2.86
950 °C	10.59	4.25	5.94	1.19	2.97	0.53	0.53	0.18
1050 °C	13.51	4.88	6.56	3.75	3.53	0.57	0.57	0.22
1100 °C	33.78	7.06	7.63	4.13	5.78	0.84	0.84	0.3

*Note:* 0 = untreated CEB, Cx = treated CEB at x% of cement, Lx = treated CEB at x% of lime, y °C = CEB fired at y °C.

**Table 9:** Designation of CEB according to their resistance.

CEB	Designation	C.E.	C.M.	Rc (MPa)	Abs. (%)
L-Tb ≥ 4 % ; C-Tb ≥ 2 % ; 2 %≤C-Gr≤6 %; 2 %≤*L*-Or≤4 %; 2 %≤C-An≤4 %; 2 %≤*L*-An≤4 %; 1050 °C-An	- OCEB 1S - OCEB 1P	- (S) - (P)	1	≥ 2	40 ≥ Abs ≥ 35 for Tb n/p 25 ≥ Abs ≥ 16 others n/p
C-Gr ≥ 8 % ; L-Gr ≥ 6 %; Gr ≥ 950 °C; C-Or ≥ 2 %; C-An ≥ 6 %; L-An ≥ 6 %; An ≥ 1050 °C	- OCEB 2S - OCEB 2P		2	≥ 4	
Tb ≥ 950 °C ; L-Gr ≥ 8 %; Gr ≥ 1100 °C; L-Or ≥ 6 %; Or ≥ 1050 °C	- OCEB 3S - OCEB 3P		3	≥ 6	
L-Tb ≥ 4 % ; C-Tb ≥ 2 % ; 2 %≤C-Gr≤6 %; 2 %≤*L*-Or≤4 %; 2 %≤C-An≤4 %; 2 %≤*L*-An≤4 %; 1050 °C-An	- CEB PN 1S or PF 1S - CEB PN 1P or PF 1P		1	≥ 2	
Tb≤1050 °C ; C-Gr ≥ 8 % ; L-Gr ≥ 6 %; Gr ≥ 950 °C; C-Or ≥ 2 %; C-An ≥ 6 %; L-An ≥ 6 %; An ≥ 1050 °C	- CEB PN 2S or PF 2S - CEB PN 2P or PF 2P		2	≥ 4	
Tb ≥ 950 °C ; L-Gr ≥ 8 %; Gr ≥ 1100 °C; L-Or ≥ 6 %; Or ≥ 1050 °C	-CEB PN 3S or PF 3S - CEB PN 3P or PF 3P		3	≥ 6	
Classification					
Locality	Batsingla	Fontsa-Touala	litakli	Fomopéa	
Mother rocks of soil	Trachybasalt (Tb)	Orthogneiss (Or)	Anatexite (An)	Biotite-hornblend granitoid (Gr)	
Nature of Soil	Clayey	Clayey+Quartz	Clay-sandy+Mica+quartz	Clay-sandy	
FAO soil classification	Fh1-ab: Humic ferrasols	Gh4-a: Humic gleysols	Nd10–3b: Dystric Nitosols	Nd10–3b: Dystric Nitosols	

## Choosing of lateritic soil origin for pavement layers or CEBs

How to choose the soil and treatment?

1st) Beforehand, determine: the availability of binders, the targeted resistance of the building (for CEBs), the targeted load-bearing class (for pavement layers) and the targeted durability of the work.
2nd) Review the literature on local geology, climate and topography.
3rd) Determine the nature of the bedrock, climate and topography.
4th) If the geological, climatic and topographic characteristics correspond to those of one of the patterns, note the pattern and continue. Otherwise stop.
5th) In the chosen model and based on the information from point 1, choose treatment method.
Note: Use pavement design guide as part of pavement layers [1].
6th) Choose the corresponding formula, table or figure and determine the components of the materials (treated or untreated, nature and dosage of the binder, type of CEB, type of pavement layer).

## Geological context

For a better relationship between the quality of construction materials (mechanical and physical quality of soil product/local construction material) and the bedrock, a review of the geological and environmental context was carried out (Table 1). The different elements considered consist of: geological formation (1^st^), age (2^nd^), topography (3^rd^), climate (4^th^), mineral and geochemical combination (5^th^).

## Geotechnical specifications



## Use of CEBs: the specification terms

The rate of absorption decreases with the increase of cement, lime or temperature. These values are used to determine the appearance, the designation and the masonry environment of the various CEBs according to the “NC 106-107: 2002–2006 [2] - reference ARS 674–675: 1996 [3] “standard. Table 9 indicates the different names of CEB according to each geological environment.

*Definitionofterms*:

*L: Lime, C: Cement, Tb: Trachybasalt, Gr: biotite and hornblend Granitoid, Or: Orthogneiss, An: Anatexite.*

*L-xr* ≥ *y% or C-xr* ≥ *y%: CEB of soil developed on x rock treated with greater than or equal to y% of lime or cement.*

*OCEB 1S: Ordinary CEB used as a non-load bearing structural element in a dry environment not subject to mechanical abrasion. Example: interior partition of a single-family house on the ground floor.*

*CEB PF 3P: CEB of thin facing used as a structural element subjected to the action of rain by lateral spraying. Example: Wall of a 3-story building of high visual quality exposed to the drizzling rain.*

*O: Ordinary; PN: Normal facing (in French “Parement Normal”); PF: Fine facing (in french “Parement Fin”);*

*C.E.: Category of environmental stress: S and P*

*S: structural element in a dry environment without risk of humification (dry environment)*

*P: structural element resistant to the action of water (action of water by lateral spraying)*

*C.M.: Category of mechanical stress: 1, 2, 3 and 4.*

*1: non-load bearing, but self-supporting structural element*

*2: structural element weakly stressed by external loads*

*3: structural element strongly stressed by external loads*

*Abs. (%): Water absorption in percentage*

*n/a: not applicable*

## Production of local building materials

Types of bricks manufactured:

- natural CEB;
- CEB treated with lime;
- CEB treated with cement,
- fired CEB.

Types of pavement layer materials performed:

- natural lateritic clayey gravel;
- lateritic clayey gravel treated with lime;
- lateritic clayey gravel treated with cement.

## Proposed formulas for determining the mode of treatment of the clay soil for CEB based on their origin

In the formulas No. 1 to 36, it should be noted that: x (Table 10) is the increase in the percentage of the hydraulic binder according to an arithmetic sequence of reason 2 %, of first term 0 %; or the increase in the firing temperature according to an arithmetic sequence of reason 50 °C, of first term 950 °C.

**Table 10:** Corresponding x value for each formula.

Corresponding x value for each formula	Soil treatment with binder or temperature: *0 = untreated CEB, Cx = treated CEB at x% of cement, Lx = treated CEB at x% of lime, y* °*C = CEB fired at y* °*C, S-0 = Untreated soil for pavement layer, S-L-i-j or S-C-i-j: "Clay soil (S) treated with (i)% of lime (L) or cement (C) after (j-4) days air curing, and 4 immersion days*.								
1	0	0	950 °C	S-0	S-0	S-0	S-0	S-0	S-0
2	C2	L2	1050 °C	S-C-2–4	S-C-2–7	S-C-2–14	S-L-2–4	S-L-2–7	S-L-2–14
3	C4	L4	1100 °C	S-C-4–4	S-C-4–7	S-C-4–14	S-L-4–4	S-L-4–7	S-L-4–14
4	C6	L6		S-C-6–4	S-C-6–7	S-C-6–14	S-L-6–4	S-L-6–7	S-L-6–14
5	C8	L8							

*Note:* x = Ci o C-i, Li or C-i, T °C

### Lateritic clay soils developed on trachybasalt (pattern I)

#### Treatment with cement (x → percentage of cement)

**Table:** 

*Correlations*	*R^2^: coefficient of determination*	*No.*
y: compressive strength of CEB in MPa		
*y* = 0.0357x^2^ + 0.1877x + 1.3	0.9191	*(1)*
y: flexural strength of CEB in MPa		
*y* = 0.045×^2^ + 0.011x + 0.284	0.9643	(2)
y: absorption of CEB in g/cm^2^/min.^0.5^		
*y* = −0.085x^2^ + 0.608x + 1.529	1	(3)

#### Treatment with lime: (x → percentage of lime)

**Table:** 

*Correlations*	*R^2^: coefficient of determination*	*No.*
y: compressive strength of CEB in MPa		
*y* = 0.0571x^2^ + 0.1271x + 1.48	0.997	(4)
y: flexural strength of CEB in MPa		
*y* = 0.0314x^2^ + 0.0514x + 0.218	0.997	(5)
y: absorption of CEB in g/cm^2^/min.^0.5^		
*y* = −0.183x + 3.3	1	(6)

#### Firing treatment: (x → temperature in °C)

**Table:** 

*Correlations*	*R^2^: coefficient of determination*	*No.*
y: compressive strength of CEB in MPa		
*y* = 8.675x^2^ – 23.105x + 25.02	1	(7)
y: flexural strength of CEB in MPa		
*y* = 0.845x^2^ – 1.975x + 4.1	1	(8)
y: absorption of CEB in g/cm^2^/min.^0.5^		
*y* = −0.168x^2^ + 0.63x + 1.549	1	(9)

 

### Lateritic clay soils developed on granitoid (Pattern II)

#### Treatment with cement (x → percentage of cement)

**Table:** 

*Correlations*	*R^2^: coefficient of determination*	*No.*
y: compressive strength of CEB in MPa		
*y* = − 0.3343 x^2^ ± 2.8237x–1.988	0.8883	(10)
y: flexural strength of CEB in MPa		
*y* = −0.0929x^2^ + 0.7951x − 0,194	0.9367	(11)
y: absorption of CEB in g/cm^2^/min.^0.5^		
*y* = 0.0145x^2^ – 0.1599x + 1.5214	0.9998	(12)

 

#### Treatment with lime: (x → percentage of lime)

**Table:** 

*Correlations*	*R^2^: coefficient of determination*	*No.*
y: compressive strength of CEB in MPa		
*y* = 0.095x^2^ + 1.149x − 1.514	0.947	(13)
y: flexural strength of CEB in MPa		
*y* = 0.0043x^2^ + 0.4523x − 0.098	0.948	(14)
y: absorption of CEB in g/cm^2^/min.^0.5^		
*y* = −0.0185x^2^ + 0.1185x + 1.074	1	(15)

 

#### Firing treatment: (x → temperature in °C)

**Table:** 

*Correlations*	*R^2^: coefficient of determination*	*No.*
y: compressive strength of CEB in MPa		
*y* = 0.775x^2^ − 1.695x + 5.17	1	(16)
y: flexural strength of CEB in MPa		
*y* = 0.08x^2^ − 0.24x + 0.73	1	(17)
y: absorption of CEB in g/cm^2^/min.^0.5^		
*y* = 0.014x^2^ − 0.089x + 1.426	1	(18)

### Lateritic clay soils developed on orthogneiss (Pattern III)

#### Treatment with cement (x → percentage of cement)

**Table:** 

*Correlations*	*R^2^: coefficient of determination*	*No.*
y: compressive strength of CEB in MPa		
*y* = −0.4371x^2^ + 3.8349x − 2.506	0.9666	(19)
y: flexural strength of CEB in MPa		
*y* = 0.0043x^2^ + 0.3903x − 0.092	0.9745	(20)
y: absorption of CEB in g/cm^2^/min.^0.5^		
*y* = 0.0173x^2^ − 0.1936x + 1.7426	0.9595	(21)

 

#### Treatment with lime: (x → percentage of lime)

**Table:** 

*Correlations*	*R^2^: coefficient of determination*	*No.*
y: compressive strength of CEB in MPa		
*y* = 0.1836x^2^ + 0.8676x − 0.282	0.949	(22)
y: flexural strength of CEB in MPa		
*y* = −0.0279x^2^ + 0.6681x − 0.408	0.939	(23)
y: absorption of CEB in g/cm^2^/min.^0.5^		
*y* = −0.083x^2^ + 0.645x + 0.01	1	(24)

 

#### Firing treatment: (x → temperature in °C)

**Table:** 

*Correlations*	*R^2^: coefficient of determination*	*No.*
y: compressive strength of CEB in MPa		
*y* = 0.225x^2^ − 0.055x + 5.77	1	(25)
y: flexural strength of CEB in MPa		
*y* = 0.115x^2^ − 0.305x + 0.72	1	(26)
y: absorption of CEB in g/cm^2^/min.^0.5^		
*y* = −0.0335x^2^ + 0.1165x + 1.254	1	(27)

 

### Lateritic clay soils developed on anatexite (pattern IV)

#### Treatment with cement (x → percentage of cement)

**Table:** 

*Correlations*	*R^2^: coefficient of determination*	*No.*
y: compressive strength of CEB in MPa		
*y* = 0.0586x^2^ + 0.4686x + 1.338	0.9868	(28)
y: flexural strength of CEB in MPa		
*y* = −0.0179x^2^ + 0.4741x − 0.17	0.995	(29)
y: absorption of CEB in g/cm^2^/min.^0.5^		
*y* = 0.0195x^2^ − 0.1353x + 1.3908	0.9917	(30)

 

#### Treatment with lime: (x → percentage of lime)

**Table:** 

*Correlations*	*R^2^: coefficient of determination*	*No.*
y: compressive strength of CEB in MPa		
*y* = 0.0857x^2^ + 0.6437x + 0.838	0.938	(31)
y: flexural strength of CSEB in MPa		
*y* = 0.1557x^2^ − 0.3243x + 0.52	0.99	(32)
y: absorption of CEB in g/cm^2^/min.^0.5^		
*y* = − 0.0497 x^2^ ± 0.3438x ± 0.6703	0.6968	(33)

 

#### Firing treatment: (x → temperature in °C)

**Table:** 

*Correlations*	*R^2^: coefficient of determination*	*No.*
y: compressive strength of CEB in MPa		
*y* = −0.59x^2^ + 3.33x − 0.55	1	(34)
y: flexural strength		
*y* = 0.02x^2^ − 0,02x + 0.18	1	(35)
y: absorption (g/cm^2^/min.^0.5^)		
*y* = −0.0175x^2^ + 0.0495x + 0.908	1	(36)

 

## Proposed formulas for determining the mode of treatment of the clay soil for pavement layers based on their origin

Among the four types of soils, those developed on trachybasalt and anatexite are fairly representative of the clayey and lateritic nature of all the soils. For these reasons, these two soils were selected to perform geological-geotechnical correlations in terms of pavement layers. Soils developed on trachybasalt is in pattern I and of class A-2–7(2) of HRB classification; while soils developed on anatexite in pattern IV and of class A-7-5 (0–12). Table 6 indicates the bearing capacity of the soil according to these patterns.

The climatic environment as defined in Table 1 is that of a hot and humid climate. The technical guide [1] and experience estimate a maximum flooding time in the rainy season of 4 days. Thus, the samples compacted with modified Proctor parameters are immersed for four days, then they are directly punched or dried for between 1 and 10 days. This allows to know the impact of variations in temperatures and precipitations on the pavement layers in lateritic clay soils.

In the formulas No. 37 to 48, it should be noted that for a fixed value of number of curing days in air after 4 curing days in water of compacted soils, x (Table 6) is the increase in the percentage of the hydraulic binder according to an arithmetic sequence of reason 2 %, of first term 0 %.

### Lateritic clay soils developed on trachybasalt (Pattern I)

y = CBR at 95% of OPM

#### 4 days of immersion in water

**Table:** 

*Correlations*	*R^2^: coefficient of determination*	*No*
Treatment with cement (x → percentage of cement)		
*y* = 4.5x^2^ + 1.7x + 33	0.9124	(37)
Treatment with lime (x → percentage of lime)		
*y* = −6.25x^2^ + 45.95x + 0.75	0.9048	(38)

 

#### 4 days of immersion in water and 3 days of dry cure

**Table:** 

*Correlations*	*R^2^: coefficient of determination*	*No.*
Treatment with cement (x → percentage of cement)		
*y* = −6x^2^ + 62.2x − 16.5	0.9607	(39)
Treatment with lime (x → percentage of lime)		
*y* = −6.25x^2^ + 64.75x − 17.25	0.9895	(40)

 

#### 4 days of immersion in water and 10 days of dry cure

**Table:** 

*Correlations*	*R^2^: coefficient of determination*	*No.*
Treatment with cement (x → percentage of cement)		
*y* = −10.25x^2^ + 93.15x − 42.75	0.9826	(41)
Treatment with lime (x→ percentage of lime)		
*y* = −6x^2^ + 77x − 30	0.9929	(42)

 

### Lateritic clay soils developed on anatexite (Pattern IV)

y: CBR at 95% of OPM

#### 4 days of immersion in water

**Table:** 

*Correlations*	*R^2^: coefficient of determination*	*No.*
Treatment with cement (x→ percentage of cement)		
*y* = −4.75x^2^ + 30.05x − 8.25	0,9412	(43)
Treatment with lime (x → percentage of lime)		
*y* = −14.25x^2^ + 90.75x − 61.75	0.9278	(44)

 

#### 4 days of immersion in water and 3 days of dry cure

**Table:** 

*Correlations*	*R^2^: coefficient of determination*	*No.*
Treatment with cement (x → percentage of cement)		
*y* = −10.5x^2^ + 64.1x − 34.5	0.9787	(45)
Treatment with lime (x → percentage of lime)		
*y* = −16.75x^2^ + 112.45x − 79.75	0.9842	(46)

 

#### 4 days of immersion in water and 10 days of dry cure

**Table:** 

*Correlations*	*R^2^: coefficient of determination*	*No.*
Treatment with cement (x → percentage of cement)		
*y* = −17.5x^2^ + 103.7x − 65.5	0.9457	(47)
Treatment with lime (x → percentage of lime)		
*y* = −18x^2^ + 129.2x − 93.5	0.9998	(48)

 

## Geology-geotechnics correlation: graphical pattern

**Fig. 1:**
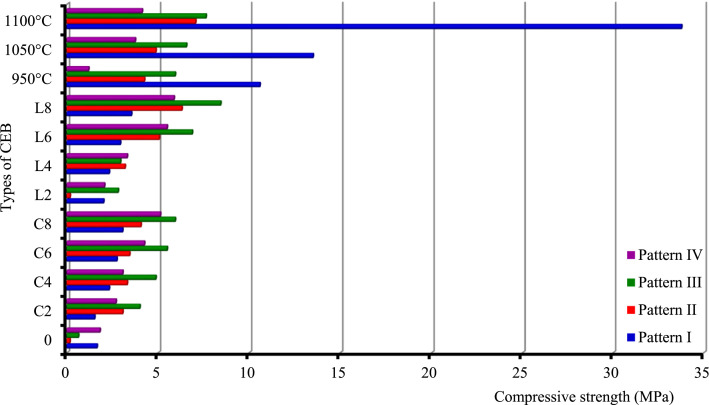
Compressive strength of CEB for the different models.

**Fig. 2:**
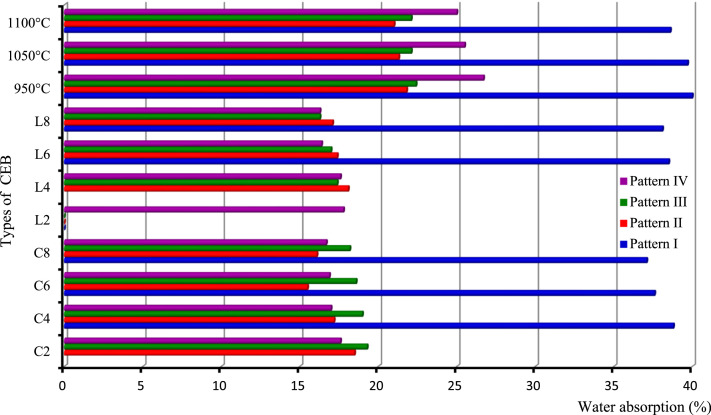
Water absorption of CEB for the different models.

**Fig. 3:**
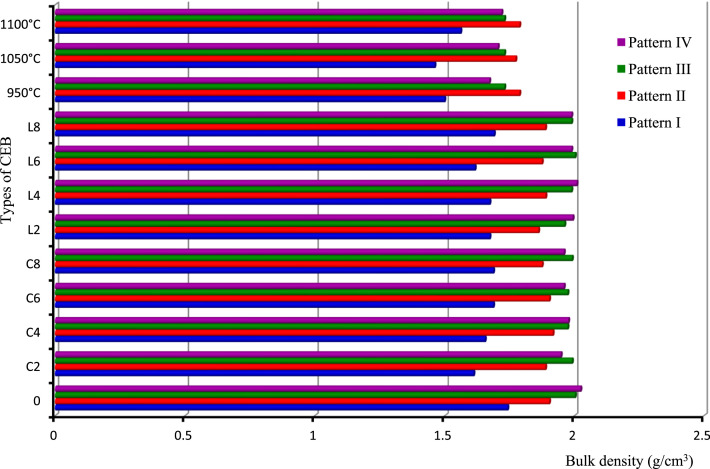
Bulk density of CEB for the different models.

**Fig. 4:**
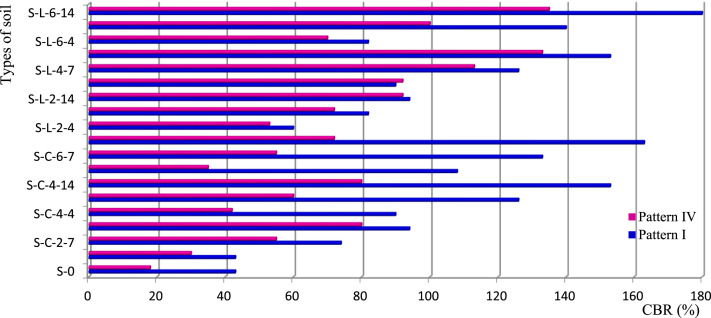
Soil bearing capacity for the different models.

Soils, products of rock weathering, are quite variable in space, which makes it difficult to represent geometrically the mechanical properties of the soils in space. Thus, this representation requires integration of the geological factors governing their implementation in order to control their interpolation [4] and [5]. The type of weathering (chemical or mechanical), the tectonics (soft or fragile), the age of the rocks (Precambrian or recent formations), the climate (temperatures, precipitations), the geomorphology (orography, hydrography) must also be considered. Furthermore, it is important to assess the local feasibility of the model so that it can be used, as in the case of a development project. The geotechnical data (Table 6, Table 7, Table 8) obtained are illustrated in bar diagrams for different patterns (Table 1, Table 2, Table 3, Table 4, Table 5). This allows a rapid and simple reading of the geotechnical abilities of the soils developed on different rocks according to its local, natural context. These geotechnical abilities are grouped into aptitude for the production of treated or untreated CEBs and soil bearing capacity in pavement layers. The different CEB formulation patterns and pavement layers are shown in Fig. 1, Fig. 2, Fig. 3, Fig. 4.

*Example: In a mountainous area with a hot and humid climate, a client wishes to construct a building with masonry elements with an average compressive strength of 5* *MPa. He also wants to create roads and various networks. He would like to know the opinion of a geotechnical engineer on the possibility of using local materials. Mapping research on the project area shows that the terrain is made up of anatexite. The latter enters in pattern IV. This rock in this climate weathers to produce soils that can be used in Civil Engineering.* Fig. 1 *indicates that CEBs treated with 8 % cement or 6 % lime have the targeted strength. The CBR value of these soils treated with 2 % of lime, after 4 days of immersion in water and 10 days of curing in air (*Fig. 4*), is greater than 80 %, an acceptable value by the design guide of pavements (CEBTP, 1984) for platforms, subgrade, foundation layer and base layer.*

## Performance of models: MAE, MSE, RSME and NS

**Table:** 

## CRediT authorship contribution statement

**Manefouet Kentsa Bertille Ilalie:** Conceptualization, Methodology, Software, Validation, Data curation, Writing – original draft, Writing – review & editing, Funding acquisition. **Kamgang Kabeyene Beyala Véronique:** Supervision, Validation, Project administration, Resources, Writing – review & editing. **Katte Yato Valentine:** Visualization, Investigation, Writing – review & editing. **Wouatong Armand:** Formal analysis, Visualization, Investigation. **Nzenti Jean Paul:** Formal analysis, Visualization, Investigation. **Ndjigui Paul Désiré:** Formal analysis, Visualization, Investigation. **Elimbi Antoine:** Formal analysis, Visualization, Investigation.

## Declaration of Competing Interest

The authors declare that they have no known competing financial interests or personal relationships that could have appeared to influence the work reported in this paper.

## Data Availability

No data was used for the research described in the article.

## Appendix Supplementary materials

Image, application 1
